# Efficient Methods for Targeted Mutagenesis in Zebrafish Using Zinc-Finger Nucleases: Data from Targeting of Nine Genes Using CompoZr or CoDA ZFNs

**DOI:** 10.1371/journal.pone.0057239

**Published:** 2013-02-22

**Authors:** Raman Sood, Blake Carrington, Kevin Bishop, MaryPat Jones, Alberto Rissone, Fabio Candotti, Settara C. Chandrasekharappa, Paul Liu

**Affiliations:** 1 Zebrafish Core Facility, Genetics and Molecular Biology Branch, National Human Genome Research Institute, Bethesda, Maryland, United States of America; 2 Genomics Core, Genome Technology Branch, National Human Genome Research Institute, Bethesda, Maryland, United States of America; 3 Disorders in Immunity Section, Genetics and Molecular Biology Branch, National Human Genome Research Institute, Bethesda, Maryland, United States of America; 4 Oncogenesis and Development Section, Genetics and Molecular Biology Branch, National Human Genome Research Institute, Bethesda, Maryland, United States of America; Hong Kong University of Science and Technology, China

## Abstract

Recently, it has been shown that targeted mutagenesis using zinc-finger nucleases (ZFNs) and transcription activator-like effector nucleases (TALENs) can be used to generate knockout zebrafish lines for analysis of their function and/or developing disease models. A number of different methods have been developed for the design and assembly of gene-specific ZFNs and TALENs, making them easily available to most zebrafish researchers. Regardless of the choice of targeting nuclease, the process of generating mutant fish is similar. It is a time-consuming and multi-step process that can benefit significantly from development of efficient high throughput methods. In this study, we used ZFNs assembled through either the CompoZr (Sigma-Aldrich) or the CoDA (context-dependent assembly) platforms to generate mutant zebrafish for nine genes. We report our improved high throughput methods for 1) evaluation of ZFNs activity by somatic lesion analysis using colony PCR, eliminating the need for plasmid DNA extractions from a large number of clones, and 2) a sensitive founder screening strategy using fluorescent PCR with PIG-tailed primers that eliminates the stutter bands and accurately identifies even single nucleotide insertions and deletions. Using these protocols, we have generated multiple mutant alleles for seven genes, five of which were targeted with CompoZr ZFNs and two with CoDA ZFNs. Our data also revealed that at least five-fold higher mRNA dose was required to achieve mutagenesis with CoDA ZFNs than with CompoZr ZFNs, and their somatic lesion frequency was lower (<5%) when compared to CopmoZr ZFNs (9–98%). This work provides high throughput protocols for efficient generation of zebrafish mutants using ZFNs and TALENs.

## Introduction

The successful completion of the Human Genome Project and the advances in sequencing technologies in the past decade have led to the sequencing of complete genomes of many species. More recently, sequencing of whole genomes and exomes has replaced traditional positional cloning approaches for the identification of disease genes and cancer-causing mutations. However, sifting through the large number of variants detected through whole genome and exome sequencing to determine the variants responsible for the phenotype under investigation is limited by our knowledge of the function of all protein coding genes. Thus, the importance of functional genomics using model organisms has been recognized as an important prerequisite for interpretation of the impact of sequence variants on the function of gene(s), leading to initiatives such as the knockout mouse project and the zebrafish phenome project [1], [2].

Zebrafish is a powerful vertebrate model organism for functional genomics due to the ease of microinjections of genetic material into embryos. Antisense oligonucleotides with a modified backbone, termed morpholinos, can be designed to block translation or splicing of specific genes. Microinjections of morpholinos or mRNA leading to transient loss or gain of function phenotypes, respectively, can be used to assess roles of genes during embryonic development [3], [4]. However, heritable genetic mutants are desirable for better understanding of gene functions, especially for larval and adult phenotypes and for generation of disease models to understand the pathophysiology of diseases. Up until recently, generation of genetic mutants relied on chemical and insertional mutagenesis approaches that caused random mutations throughout the genome [5], [6]. Of these, chemical mutagenesis using N-ethyl-N-nitrosourea (ENU) followed by forward (phenotype-based) and reverse (gene-based, also termed TILLING for Targeting Induced Local Lesions IN Genomes) screens became popular among the zebrafish community [7], [8], [9], [10]. Recent application of next-generation sequencing technology to TILLING is predicted to generate mutations in thousands of genes in the coming years [11]. However, there are several limitations to identification of genetic mutants for all genes using TILLING [12]. First, the odds of finding knockout mutations are dependent upon the size and sequence of the coding exons, the mutation frequency per genome induced by the ENU-treatment, and the number of ENU mutagenized genomes being screened. Therefore, genes with short open reading frames and/or short exons are not ideal for TILLING. Second, the recovery of the identified mutation from frozen sperm or the founder fish may fail due to poor quality of the frozen sperm, death of the founder fish, or difficulty with breeding. Third, each founder fish typically carries hundreds of ENU-induced mutations, which may slow and/or complicate phenotype analysis of the mutant fish.

Recently, several studies have demonstrated that zinc-finger nucleases (ZFNs) and transcription activator-like effector nucleases (TALENs) can be used to specifically target a gene to generate heritable knockout mutants in zebrafish [13], [14], [15], [16], [17], [18], [19], [20], [21], [22]. ZFNs and TALENs are artificially produced hybrid proteins that allow targeting to a desired site in the genome by combining the sequence specificity feature of zinc-finger proteins (ZFPs) and transcription activator-like effector proteins (TALEs), respectively, with the non-specific endonuclease activity of *Fok1* nuclease [23], [24], [25]. Double strand breaks in DNA caused by *Fok1* nuclease are repaired by the endogenous error-prone non-homologous end joining repair pathway, leading to insertions or deletions (in/dels) at the cut site [26]. In both approaches, two units, termed left and right zinc fingers or TAL-effector arrays, are designed to provide sequence specificity and fused to the *Fok1* nuclease. Heterodimeric subunits of *Fok1* can be used to increase specificity by minimizing off-target effects caused by binding of two left or two right ZFN or TALEN units [14], [27], [28]. The length of the spacer between the left and right units, where double strand break occurs, varies from 5–7 base pairs (bp) for ZFNs and is more flexible, ranging from 10–21 bp, for TALENs [29], [30].

There are various options for designing and assembling ZFN and TALEN pairs for specific genes. Ready to use expression plasmids can be purchased from commercial sources (e.g. ZFNs from Sigma-Aldrich and TALENs from Life Technologies, Transposagen, and Cellectis) or assembled in the laboratory using publicly available resources, such as OPEN, CoDA, and modular-assembly for ZFNs and Golden Gate, FLASH, Iterative capped assembly and other methods for TALENs [17], [18], [22], [24], [30], [31], [32], [33], [34], [35], [36], [37]. While assembling ZFNs through OPEN and modular assembly are technically challenging and labor-intensive, CoDA approach is straightforward requiring two easy steps: 1) computational analysis of the target gene to identify potential ZFP binding sites, 2) synthesis and cloning of the ZFP coding sequences into appropriate expression vectors containing the heterodimeric *Fok1* nuclease domain. Therefore, we used CompoZr (Sigma Aldrich) and CoDA approaches for designing ZFNs for our study. Here we present our data on their performance in targeting of nine genes in zebrafish. In the process, we have generated multiple loss-of-function alleles for seven genes and developed high throughput protocols for performing ZFN targeting in zebrafish. We recommend using these protocols for gene targeting independent of the source of targeting nuclease (ZFNs or TALENs) used.

## Materials and Methods

### Ethics Statement and zebrafish lines used

This study was approved by the National Human Genome Research Institute Animal Care and Use Committee, OLAW Assurance # A-4149-01 under protocol # G05-5. Zebrafsih were housed in accordance with the Guide for the Care and Use of Laboratory Animals of National Institutes of Health in an AAALAC (Association for Assessment and Accreditation of Laboratory Animal Care) accredited facility. All zebrafish handling and breedings and were performed in accordance with the methods published in the Zebrafish Book [38]. All efforts were made to minimize suffering and pharmaceutical grade buffered tricaine was used for euthanasia as recommended in the Zebrafish Book. All experiments were performed in Wild-type fish of genetic strains EK (Ekkwill), AB and Tu (Tubingen).

### Design and assembly of CompoZr ZFNs

CompoZr ZFNs were designed and characterized by Sigma-Aldrich (St. Louis, MO). Briefly, for each target gene, 16 ZFN pairs were identified with recognition sequences within the first half of the coding sequence. The ZFN pairs were evaluated for activity at the target site using either the surveyor assay (Transgenomic, Omaha, NE) of transfected zebrafish fibroblast cell line SJD.1 or the yeast *MEL1* reporter assay [16]. We received mRNA for the ZFN pair with the highest activity (Table 1) and plasmid DNA for one to two additional pairs that ranked at numbers 2 and 3. Expression vectors with improved obligate heterodimeric *Fok1* variants, termed ELD/KKR (all pairs listed in table 1 except mmachc-E2) and NELD/CKKR (mmachc-E2) were used [39]. Except for ZFNs targeting *mmachc*, the pair with highest *in vitro* activity for each gene also demonstrated activity at the target site in zebrafish, so the additional pairs were not evaluated.

**Table 1:** Target sequences and in vitro activity of CompoZr ZFNs.

Gene	Accession number	Target exon	Target sequences (Left ZFN - Spacer - Right ZFN)^a^	*in vitro* activity^b^
*ak2*	ENSDARG00000005926	1	aCGATACCGTCTCCGGTAT*acgga*AAGGCATACGGGc	131.7%
*cbfb*	ENSDARG00000040917	3	gTTCTTCCCAGCC*aacct*TCATGGGGATCAGCGg	2.4%
*eomesb*	ENSDARG00000019428	2	gTACAGCGGGCAGACCGGA*gccgtg*TACGCCGGGTCGGATGGGt	155.1%
*igf2bp2a*	ENSDARG00000003421	1	cCGGCTATGCcTTCGTG*gacttc*CCCGACcaGAACTGGGCGATt	150.8%
*mmachc*	ENSDARG00000043877	4	cACCCTCAGCTGGgCGGCTG*gtttgc*GATCCG**T**GCGCTgCTGGTGt	114.1%
		E3/I4	aGACATCACACATCC**A**CAAT*ccgcc*ATGGGGAGAGAAGGTCAGt	52.1%
		2	tCACCGCTGCTCA*tcatct*GCAGTATCCAGCAGACa	120.4%

a : Spacer sequences are shown in lower case letters in italics. Bold letters denote polymorphic sites. Underlined letters denote the splice site. Lower case letters denote nucleotides flanking the left and right ZFN recognition sequences and gaps between adjacent ZFPs.

b : *in vitro* activity was measured by Mel1 reporter assay and required at >50% except for *cbfb* where zebrafish SJD.1 cells were transfected and activity was measured by surveyor assay (>1% required).

### Design and assembly of CoDA ZFNs

For assembling ZFNs using CoDA, we used the ZiFit Software program available at http://zifit.partners.org/ZiFiT/ to identify potential ZFN target sites within the first half of the coding sequence [40]. To identify potential off-target binding sites, we ran a custom script that can identify every match between a query and target sequence, allowing for a specified number of mismatches. Here, we searched for all matches between the ZFN recognition sequences and the zebrafish genome, allowing for upto 8 mismatches. Next, we performed polymorphism analysis of the exons containing potential ZFN target sites by PCR amplification and sequencing of genomic DNA from 8 adult fish of the line we will subsequently use for the injections to determine any single nucleotide polymorphisms (SNPs) affecting the ZFN binding sites. Therefore, the final two pairs of CoDA ZFNs (Table 2) for each gene were selected after filtering through the potential target sites for 1) location within the open reading frame, 2) minimal genome-wide hits in the ZFN binding sites and 3) absence of SNPs.

**Table 2:** Target sequences for CoDA ZFNs: 2 pairs were selected for each gene.

Gene	Accession number	Target exon	Target sequences (Left ZFN - Spacer - Right ZFN)^a^
*cmet*	ENSDARG00000070903	1	tCTCACCGTC*caacgc*GAAGGTGGCa
		3	cAACGGCTCC*ttatt*GTTGATAACa
*hint3*	ENSDARG00000074286	1	cACCGACACC*tgaga*GCTGTATAGc
		E1/I2	cTTCTACACA*gcgtaag*TGTGCTGTCa
*kctd7*	ENSDARG00000061580	1	tGGCAACATC*acgggt*GAGGAGGTTc
		2	gGTCATCCCC*ttgaat*GTAGGAGGAa
*stat3*	ENSDARG00000022712	3	gTACAGCCGC*ttcct*GCAGGAGAAc
		6	cAGCAGCCAC*cagaca*GAAGATGTCt

a : Spacer sequences are shown in lower case letters in italics. One nucleotide flanking the left and right ZFN recognition sequences is shown in lower case letters. Splice site in *hint3* ZFN pair 2 is underlined.

Nucleotide sequences coding for the left and right ZFP arrays of selected pairs including the required restriction enzyme recognition sequences were downloaded from ZiFit and synthesized as minigenes (Integrated DNA Technologies, Coralville, IA). The following expression vectors, with EL/KK heterodimeric *Fok1* variants as described in [27], were obtained from Addgene: pMLM290 (Addgene ID 21872), pMLM292 (Addgene ID 21873), pMLM800 (Addgene ID 27202), and pMLM802 (Addgene ID 27203). Cloning into appropriate expression vectors was performed as follows: minigene DNA for ZFN arrays with 5 and 6 bp spacers were digested with *BamH1* and *Xba1* and ZFN arrays with 7 bp spacer were digested with *Xba1* and *Not1* and purified by gel electrophoresis using QIAquick Gel Extraction Kit (Qiagen, Valencia, CA). Simultaneously, the expression vectors were digested with the same set of restriction enzymes, purified using MinElute PCR Purification Kit (Qiagen, Valencia, CA) and treated with Antarctic Phosphatase (New England Biolabs, Ipswich, MA). Ligations were preformed as follows: the left finger arrays were ligated into pMLM290 (5 bp and 6 bp spacer) or pMLM800 (7 bp spacer) and the right finger arrays into pMLM292 (5 bp and 6 bp spacer) or pMLM802 (7 bp spacer). The Qiagen mini-prep kit was used to extract plasmid DNA for screening of clones and after sequence verification plasmid DNA was extracted using the maxi-prep kit (Qiagen, Valencia, CA).

### mRNA synthesis and injections

mRNAs encoding the left and right zinc finger arrays for each CoDA ZFN pair were synthesized using mMessage mMachine T7 Ultra kit (Life Technologies, Grand Island, NY) as previously described [17]. For both CompoZr and CoDA ZFNs, mRNAs encoding the left and right arrays for a given pair were mixed in equimolar amounts and injected at multiple doses into zebrafish embryos at 1-cell stage using standard microinjection technology [38]. Embryos were then screened for toxicity at 24 hours post fertilization (hpf) and the doses with >50% dead and/or morphologically deformed embryos were considered toxic. Injections were repeated at the appropriate dose based on the toxicity test for determining ZFN efficiency and growing to adulthood for founder screening.

### Determination of ZFN efficiency by somatic lesion analysis

To determine ZFN activity at the target site, eight to ten embryos injected with the appropriate dose were collected at 48 hpf. DNA was extracted using the Extract-N-Amp Tissue PCR Kit (Sigma-Aldrich, St. Louis, MO). Extracted DNA was diluted 1∶10 with ultra-pure water and 2 µl were used as template in a standard 50 µl PCR reaction using AmpliTaq-Gold (Life Technologies, Grand Island, NY). Gene-specific primers were designed to amplify 230–350 bp fragments encompassing the ZFN binding site, preferably placing it in the middle of the amplicon and tailed with M13F and M13R sequences (Table S1). The PCR conditions were as follows: 12 min denaturation at 94°C; 35 cycles of 94°C for 30 sec, 57°C for 30 sec, and 72°C for 30 sec; and 10 min final extension at 72°C. PCR products were purified using MinElute PCR Purification Kit (Qiagen, Valencia, CA) and cloned into pCR4-TOPO vector (Life Technologies, Grand Island, NY) using 2 hours of incubation for ligation instead of the recommended 5 minutes incubation to get sufficient colonies. Colony PCR was performed as follows: 50 µl of PCR master mix containing everything except template DNA was dispensed into each well of a 96-well plate. Individual colonies were picked with sterile P-20 pipette tips and dipped into the master mix followed by PCR as described above. Three microliters of PCR products were sequenced with M13F or M13R primer and big-dye v3.1 sequencing mix (Life Technologies, Grand Island, NY) after removal of unused primers and nucleotides with Exo-SAP-IT (Affymetrix, Cleveland, OH). Sequence analysis was performed using software package Sequencher, version 5.0 (Gene Codes, Ann Arbor, MI).

### Founder screening and identification of heterozygous adult fish by fluorescent PCR

Each potential founder fish was crossed with a Wild-type fish and 60 embryos were harvested at 48 hpf as 15 pools of 4 embryos each in two columns of a 96-well PCR plate. DNA extraction was performed using Extract-N-Amp kit (Sigma-Aldrich, St. Louis, MO) as described above. To avoid cost associated with labeling of individual primers we used a mixture of M13F-tailed (5′- TGTAAAACGACGGCCAGT-3′) gene-specific forward primers and a common fluorescently labeled M13F primer (6-FAM, HEX or TAMRA) in the PCR mix (Table S1). Furthermore, for efficient genotyping without stutter peaks, we used PIG-tailed (5′- GTGTCTT-3′) gene-specific reverse primers [41] (Table S1). PCR reactions were set-up in 6.5 µl final volume using above-mentioned three primers in equimolar ratios and AmpliTaq-Gold (Life technologies, Grand Island, NY) and PCR was performed as described above.

PCR products were processed for fragment separation by capillary electrophoresis on either a Genetic Analyzer 3100 using POP-4 polymer or a Genetic Analyzer 3130xl using POP-7 polymer. The ROX400 size standard (Life technologies, Grand Island, NY) was run as an internal size marker by adding 10 µl of 1∶25 mix of ROX400 and HiDi-formamide to 1.5 µl of PCR product. Samples were denatured at 95°C for 5 minutes and run on the Genetic Analyzer. Data were analyzed for allele sizes and peak heights using the GeneScan and Genotyper software of GeneMapper package (Life technologies, Grand Island, NY). Similarly, adult fish from germline transmitting founders were genotyped by fluorescent PCR on DNA extracted from fin clips.

### Determination of mutant allele sequences

DNA from the embryo pools showing mutant peaks during founder screening were amplified and cloned into pCR4-TOPO vector (Life technologies, Grand Island, NY). Colony PCR, sequencing and analysis were performed on 16–24 clones as described above to determine the exact sequence of the mutant alleles. Sequence analysis was performed using Sequencher, version 5.0 (Gene Codes, Ann Arbor, MI).

## Results

### Target genes and design of ZFNs

**Figure 1:**
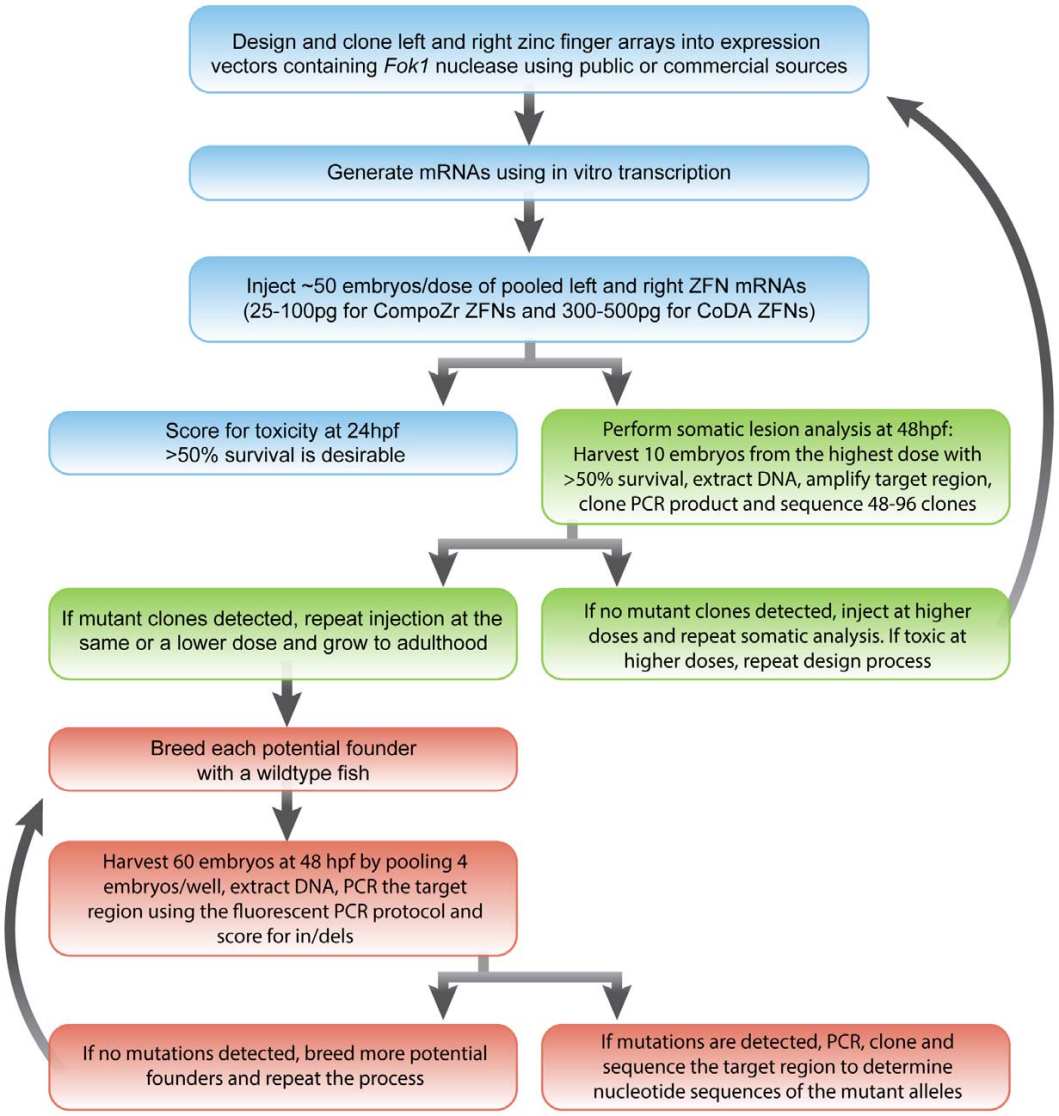
Flowchart of step-by-step experimental procedures for generating mutant zebrafish lines with ZFNs. Steps involving ZFN design, mRNA injections, and toxicity assessment are shown in blue color, efficiency testing using somatic lesion analysis in green color and founder screening steps in orange color.

The overall strategy of our experimental procedures is depicted in Figure 1. We used CompoZr ZFNs for five genes (Sigma-Aldrich) (Table 1) and CoDA ZFNs for four genes (Table 2). Based on the predicted success rate of ∼50% for CoDA ZFNs [35], we assembled two ZFN pairs for each of the four genes targeted by the CoDA approach (Table 2). Given the high degree of DNA sequence heterogeneity in zebrafish [42], we performed sequence analysis of all potential target regions identified by the CoDA approach for polymorphisms in the fish line we would use for subsequent targeting to choose two pairs that were not affected by SNPs.

### Evaluation of ZFN activity at target sites by somatic lesion analysis

For each ZFN pair, we performed injections at multiple doses and evaluated toxicity at 24 hpf (Figure S1). We harvested eight to ten embryos from the highest dose for somatic mutation analysis (Table 3). Five out of seven CompoZr ZFN pairs and two out of six CoDA ZFN pairs showed evidence for activity at the target site. CompoZr ZFN pairs targeting *eomesb* and *igf2bp2a* were highly efficient in generating a variety of in/dels, producing >90% of clones with mutations. Overall, the somatic lesion efficiency ranged from none (*hint3* and *stat3*) to <5% for CoDA ZFNs (*kctd7* and *cmet*) and from 9 to 98% for CompoZr ZFNs. While initially designed CompoZr ZFN pairs targeting exons 3 and 4 of the *mmachc* gene failed to show any somatic lesions, a new pair targeting exon 2 resulted in mutations in 17% of the clones.

**Table 3:** Efficiency of the tested CompoZr and CoDA ZFNs.

Gene and ZFN location	Somatic lesion frequency in injected embryos		Frequency of germline transmitting founders	
	mRNA Dose (pg)	Mutant clones % (mutant clones/# sequenced)	mRNA Dose (pg)	Founders % (transmitting founders/# screened)
**CompoZr ZFNs**				
*ak2*	110	26% (9/34)	27	85% (12/14)
*cbfb*	30	9% (2/21)	30	23% (7/30)
*eomesb*	92	98.5% (65/66)	32	100% (7/7)
*igf2bp2a*	100	90.5% (67/74)	25	1005 (5/5)
*mmachc:E4*	75–167	0/160	50–100	0/22
*mmachc:E3*	35–50	0/86	35	0/9
*mmachc:* E2	110	17% (16/93)	27	33% (6/18)
**CoDA ZFNs**				
*cmet:* E1	500	4.5% (2/45)	400–500	25% (3/12)
*kctd7:* E1	500	2.5% (2/78)	400–500	10% (3/30)
*hint3:* E1	400–500	0/132	400	0/7
*hint3:* E1/I2	400–500	0/170	500	0/4
*stat3:* E3	82–500	0/85	82	0/6
*stat3:* E6	82–500	0/249	Not done	

We do not know the exact reason for failure of the CompoZr ZFN pairs targeting exons 3 and 4 of *mmachc*. Interestingly, a polymorphic site within the ZFN binding sites was detected in both ZFN pairs (shown in bold in table 1). However, our subsequent efforts to inject in the fish of other strains carrying the right allele also failed to cause mutations at the target site, ruling out SNPs as the reason for inactivity of these two ZFN pairs. Of the eight CoDA ZFN pairs, four pairs targeting *stat3* and *hint3* did not yield any mutant clones at multiple doses of injected mRNA (Table 3). For the failed ZFN pairs, one possible explanation could be that their efficiency is below the detection threshold of our assay. Therefore, for practical purposes we considered them inactive.

### Founder screening and efficiency of germline transmission

**Figure 2:**
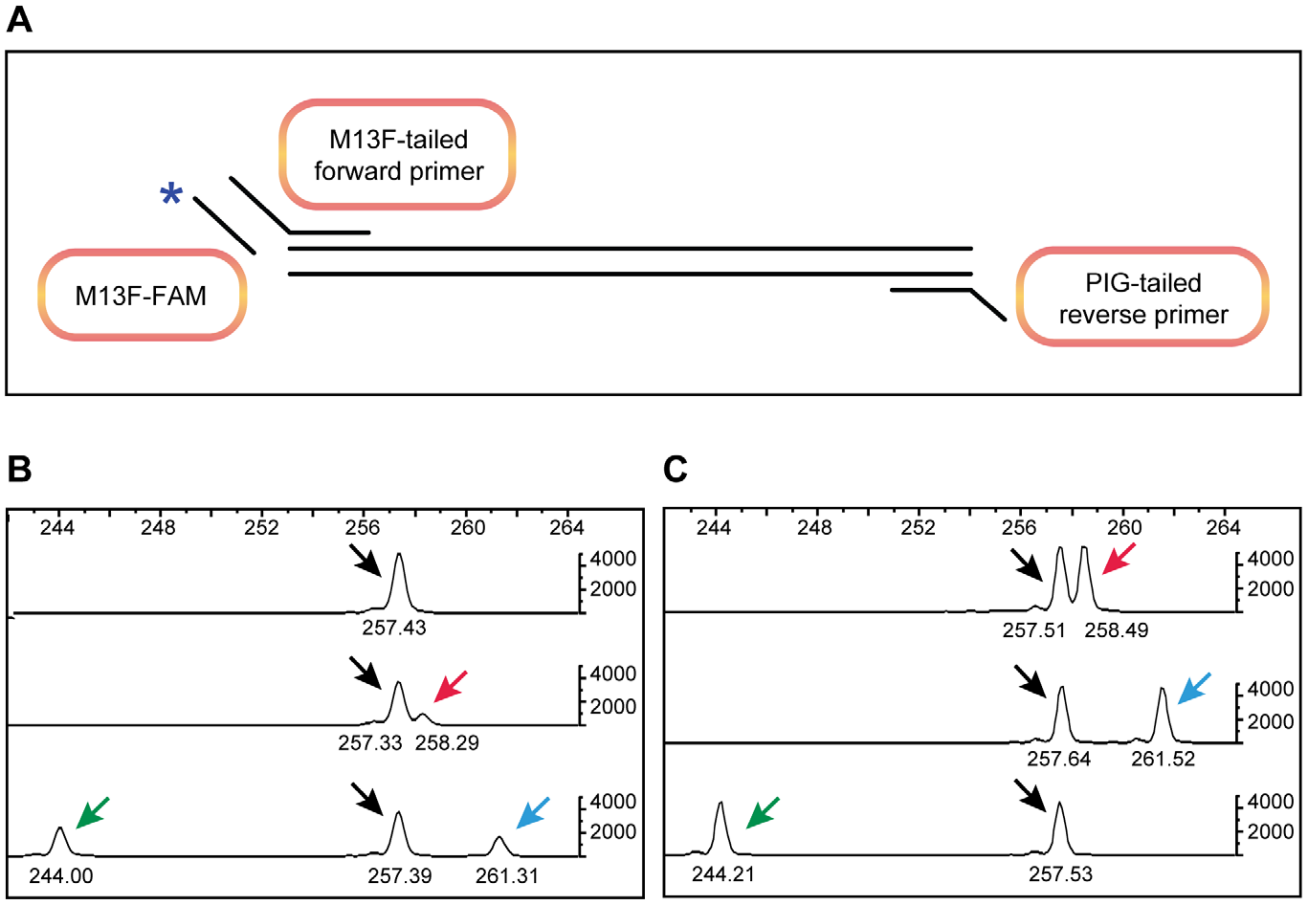
Fluorescent PCR strategy and examples of mutant peaks from founder screening and F1 genotyping. A) Schematic of the fluorescent PCR strategy. Target region is shown as two black lines. Forward primers are denoted as FAM (blue star) -labeled M13F primer (M13F-FAM) and gene-specific primer with M13F tail (M13F-tailed forward primer). Reverse primer is denoted as the gene-specific part and the PIG-tail sequence (PIG-tailed reverse primer). B and C) Founder screening for *ak2* using 4 pooled embryos per well (B) and genotyping of heterozygous adults from F1 progeny of the corresponding transmitting founders (C). Fragment size scales are shown on the top and each fragment's size is marked underneath the peak. Vertical scale marks the intensity of the peaks. Black arrows mark the 257 bp peak corresponding to the Wild-type allele observed in all samples. The top panel in B is a Wild-type control DNA sample, the middle panel is a founder transmitting a 1 bp insertion mutation (258 bp, marked by a red arrow) and the bottom panel shows a founder transmitting two mutations, a 13 bp deletion (244 bp, marked by a green arrow) and a 4 bp insertion (261 bp, marked by a blue arrow). In C each panel shows a heterozygous adult zebrafish and color-matched arrows mark the mutant peaks.

To minimize the off-target effects for ZFNs with high frequency of somatic lesions (>10%), we performed injections at lower doses (up to one quarter of the initial dose) and grew injected embryos to adulthood for founder screening (Table 3). We devised a cost-effective and efficient founder screening strategy using pooled embryos and fluorescently labeled M13F primer and gene-specific primers with specific tails (Figure 2A). We reasoned that by pooling 4 embryos/well, we could screen 60 embryos from each putative founder fish, enabling us to detect mutations transmitted at 1.6% (1 in 60 embryos tested) or higher efficiency. This strategy allowed us to screen 6 putative founders in a 96-well plate. As expected, majority of the pools showed peaks of expected size indicating that they carried Wild-type alleles only (Figure 2B, top panel). For embryo pools containing mutant embryos, we detected peaks of different sizes at 1/8^th^ the intensity of the Wild-type peak indicating that one of the four embryos in the pool carries a heterozygous mutation (Figure 2B, middle panel); or peaks with higher intensity indicating that multiple embryos in the pool carry the mutation (Figure 2B, bottom panel). We also detected multiple peaks indicating embryos in the pool carry different mutations (Figure 2B, bottom panel). Genotypes of corresponding heterozygous adults recovered from out-cross of these founders are shown in Figure 2C.

Overall, for ZFNs with high frequency of somatic lesions, almost all injected fish transmitted one or more mutations (up to nine) to their progeny, despite the fact that a lower mRNA dose of ZFNs was injected for founder screening (Table 3). For ZFNs that showed lower somatic lesion frequency (<5%), e.g. CoDA ZFNs, 10–25% of screened adults were identified as germline transmitting founders. Therefore, in most cases, screening of one plate of embryo pools from male founders and one plate from female founders was enough to identify two independent founders with loss of function mutations. For the ZFN pairs that failed to generate somatic lesions, we did not identify any germline transmitting founders either. However, we screened only a small number of putative founders and it is reasonable to assume that their efficiency is lower than our detection limits.

### Effective mRNA dose

Based on our data from somatic lesion analysis and founder screening, the effective mRNA dose range was determined to be 25–110 pg for CompoZr ZFNs and 400–500 pg for CoDA ZFNs (Table 3). Toxicity to injected embryos increased at doses higher than 500 pg. Although we did not test CompoZr ZFNs at mRNA doses lower than 25 pg, given their efficiency in generating founders with multiple mutations, we recommend evaluating at least one dose lower than 25 pg for CompoZr ZFNs.

### Range of ZFN-induced mutations at the target site

**Figure 3:**
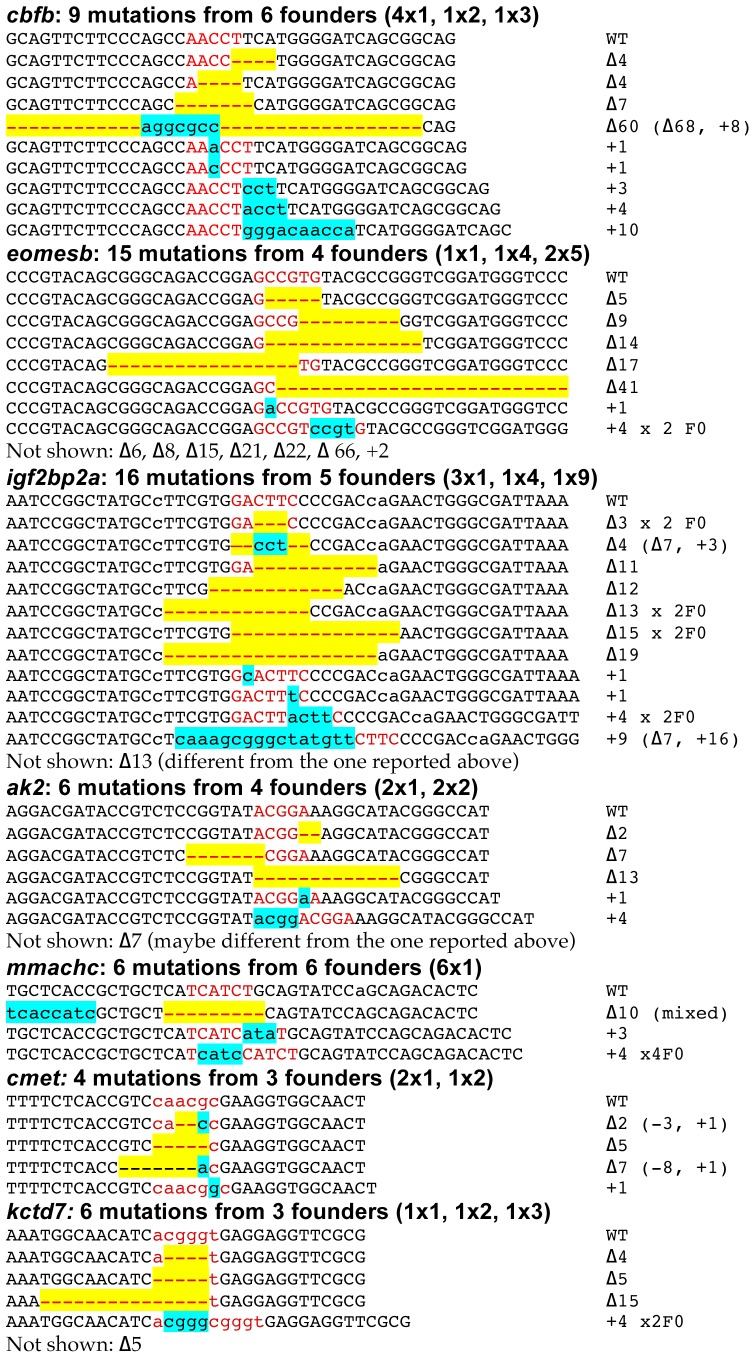
List of all mutations identified by founder screening and genotyping of F1 adults. For each target gene, the total number of mutations, total number of germline-transmitting founders, and number of mutations transmitted by each founder are given. For example, for *cbfb* 4×1, 1×2, 1×3 indicates 4 founders each transmitted a single mutation, one founder transmitted two mutations and another founder transmitted three mutations, for a total of 9 mutations. In all cases, the Wild-type sequences with spacer marked in red are shown at the top followed by the sequences of the mutant alleles. Deletions are marked by red dashes highlighted in yellow and insertions by lower case letters highlighted in blue color. The nature of each mutation is indicated on the right side of the sequence, Δ indicates deletion, + indicates insertion. In some cases, identical mutations were transmitted by multiple founders and these are indicated by x # Fo (meaning times # of founders). Finally, additional mutations whose sequences were not determined are listed at the bottom for each gene.

We observed insertions and deletions ranging from 1 bp to >60 bp as determined by the allele sizes in fluorescent PCR for the seven ZFN pairs that demonstrated activity by somatic lesion analysis. The exact sequences of mutant alleles were determined by sequencing clones from PCR products from the embryo pools with mutant peaks in fluorescent PCR. Most of the mutations were insertions or deletions, while a small number of them were complex mutations involving both insertion of a few random nucleotides and a deletion (Figure 3). The most common mutation was an insertion leading to a 4 bp duplication at the cut site (Figure 3). Interestingly, multiple independent founders transmitted identical 4 bp insertions in four of the seven genes, for example, 4 independent founders for *mmachc* transmitted the same CATC insertion. We also observed that larger deletions (>10 bp) were more frequent than larger insertions.

### Efficiency of recovery of heterozygotes from F1 generations

As shown in figure 2C, we performed fluorescent PCR to genotype adult zebrafish from the F1 generation. Based on our founder screening strategy, we would be able to detect mutations with a minimum of 1.6% of the F1 generation to be heterozygous for a given mutation. In most cases recovery of mutations in the F1 generation was at a higher frequency than expected from the frequency in embryo pools (Table 4). One possible explanation is that we underestimated the transmission frequency from embryo pools assuming only one embryo was heterozygous in a given pool with mutant peaks. Furthermore, additional mutations were often detected at low frequencies (0.5–2%) in the F1 progeny, especially when a large number of fish were genotyped to recover the desired mutations. Overall, the transmission frequency in embryo pools was used as a guide to determine the number of F1 adults to be genotyped for recovery of the desired alleles.

**Table 4:** Rate of germline transmission of ZFN-induced mutations.

Gene	# of germline transmitting founders^a^	Mutations detected in embryo pools		Mutations detected in heterozygous adults	
		# of mutations	Range of transmission frequency^b^	# of mutations	Range of transmission frequency
**CompoZr ZFNs**					
*ak2*	4	6	3.5–13%	6	3.5–31%
*cbfb*	6	6	2.5–11%	8	4–25%
*eomesb*	4	14	2–20%	15	1–24%
*igf2bp2a*	2	6	5–20%	10	0.5–17%
*mmachc*	6	6	2–8%	3	12.5–19%
**CoDA ZFNs**					
*cmet*	3	4	3–12%	4	1–16%
*kctd7*	2	3	3–17%	3	4–7%

a : Counted only the founders where progeny was analyzed by both methods.

b : Calculated assuming only one embryo in the pools with mutant peaks is heterozygous.

## Discussion

Zebrafish provide an *in vivo* vertebrate animal model system for functional studies of genes of unknown function. Recent advances in targeted mutagenesis using ZFNs and TALENs have overcome the limitations of previously available reverse genetics techniques in zebrafish that relied on random mutagenesis with chemical or retroviral methods. In the last couple of years, a plethora of cheaper and faster methods to design and assemble ZFNs and TALENs have been developed, thus making the technology accessible to all zebrafish laboratories. However, the design and acquisition of the appropriate nuclease is only the first step in using this technology to generate mutant fish. It would be of great benefit to develop general guidelines for successful application of ZFNs and TALENs to large-scale zebrafish knockout projects.

One of the critical factors in successful targeting of desired loci using ZFNs is the amount of mRNA injected per embryo. While lower mRNA dose may not cause any mutations at the target site, higher dose can lead to increased mutagenesis at the off-target sites in the genome [43]. The effective mRNA dose is often determined empirically by performing evaluation of injected embryos for toxicity at multiple doses. In the published reports, the effective mRNA dose varied by more than 1000-fold, ranging from 5 pg to 7 ng [16], [17], [19], [22], [33]. We observed high toxicity at doses higher than 500 pg and therefore, tested most ZFNs at 25–500 pg range. Based on our data, at least five-fold higher dose of mRNA is required for CoDA ZFNs when compared to CompoZr ZFNs. One noticeable difference between them is the specificity provided by the length of their recognition sequences. CoDA ZFNs are limited to 3 zinc-fingers on either side, giving a specificity of 9 bp as monomers. On the other hand, in most cases CompoZr-ZFNs consisted of 4–6 zinc-fingers on either side, thus recognizing 12–18 bp sequences as monomers. It is possible that ZFNs with shorter recognition sequences have increased chances of binding to other sites in the genome as monomers, thus requiring increased mRNA dose to induce mutations at the target site. Another possible explanation for these differences could be the use of improved obligate heterodimeric *Fok1* variants (ELD/KKR) for CompoZr ZFNs as opposed to EL/KK *Fok1* variants used for CoDA ZFNs. Doyon and colleagues [39] demonstrated that ELD/KKR *Fok1* variants worked better in inducing somatic lesions than the EL/KK variants when used with the same ZFPs. For a better comparison, expression vectors with identical *Fok1* variants with improved activity, such as Sharkey [44], or ELD/KKR [39] should be used with both CompoZr and CoDA ZFPs. Similarly, ZFNs targeting the same genomic loci should be assembled using CompoZr and CoDA platforms, with subsequent evaluation of their targeting efficiencies. This would rule out any differences in their performance due to the differences in the chromatin structure and accessibility of the target sites.

It is critical to evaluate the designed ZFNs by somatic lesion analysis before proceeding to growing the injected embryos to adulthood for founder screening. This is often done by surveying 8–10 injected embryos by PCR, followed by cloning and sequencing of large number of clones. Based on our results, sequencing of 48 clones is sufficient for highly efficient ZFNs (efficiency of >25%). Therefore, one can start with sequencing of 48 clones and sequence more if no mutant clones are identified. This decision can also be made based on the source of ZFNs used, as CoDA ZFNs have lower efficiency compared to CompoZr ZFNs.

Previous reports have compared the performance of CoDA ZFNs with ZFNs assembled through OPEN protocol and TALENs [20], [35]. Roughly half of the CoDA ZFNs pre-selected by their *in vitro* activity at the target site using bacterial two- hybrid (B2H) assay were found to be active in zebrafish [35]. We used two CoDA pairs per gene without screening with B2H assay for their activity. Both pairs for *stat3* and *hint3* failed to introduce somatic mutations in injected embryos. Since one of the 2 ZFN pairs worked for *cmet* and *kctd7*, we did not extensively test the other pair for these two genes. Therefore, in our study two out of six CoDA ZFNs (∼33%) were successful at introducing somatic mutations in zebrafish. This success rate is higher than the observed (18%) success rate and comparable to the estimated success rate (28%) for non-selected CoDA ZFNs [20]. However, one must be cautious in interpreting the success rates of different studies as the threshold of detection methods varies between these studies.

A major concern in using ZFNs and TALENs is their potential for binding to other sites in the genome that may have some sequence similarity with the target site, leading to background mutations [19], [45]. Based on our data, for most ZFNs with >1% somatic lesion efficiency, it is relatively easy to identify at least two independent founders transmitting different in/dels leading to loss-of-function mutations. Therefore, we recommend generation of at least two mutant alleles from two independent founders for each gene for phenotypic analysis. To save time and resources, compound heterozygotes can be investigated instead of homozygotes for the mutations separately.

In conclusion, we have generated multiple mutant zebrafish lines for seven genes that would help in delineating their function during development and disease. In parallel, we have developed methods that use equipment and reagents readily available to most zebrafish laboratories for performing targeted mutagenesis of their desired genes. Our improved method for somatic analysis using colony PCR can be employed for quick evaluation of activity of a given pair of ZFNs, thus determining whether to proceed with growing the injected fish for founder screening or inject at lower or higher mRNA dose or design another ZFN pair. Our fluorescent PCR protocol for founder screening is high throughput, robust, and cost-effective as it eliminates the need for labeled primers for each target and allows for easy identification of the 1 bp in/dels by elimination of the stutter peaks that were observed by Foley and colleagues [17]. Although we have only used ZFNs in our study, these protocols should also be applicable to TALENs as the subsequent steps are identical in both cases. Thus our improved methods can be applied to streamline the process of generating mutant zebrafish lines for large-scale knockout projects using ZFNs and TALENs.

## Supporting Information

Figure S1 **Toxicity data for CompoZr ZFNs (A) and CoDA ZFNs (B).** ZFN ID's and injected doses are listed on the X-axis and percentage of normal (WT = green), deformed (Monster = red) and dead (blue) embryos at 24 hpf are shown on the Y-axis. Numbers in the bar graphs denote the actual numbers of embryos in each category.
(TIF)

Table S1 **Sequences of PCR primers used to amplify genomic DNA for somatic lesion analysis, founder screening and F1 adult genotyping.**
(XLSX)
