# Supplementary figures and images for: Efficient Methods for Targeted Mutagenesis in Zebrafish Using Zinc-Finger Nucleases: Data from Targeting of Nine Genes Using CompoZr or CoDA ZFNs
Source: PLoS One. 2013 Feb 22;8(2):e57239. doi: 10.1371/journal.pone.0057239 (PMC3579846; doi:10.1371/journal.pone.0057239)

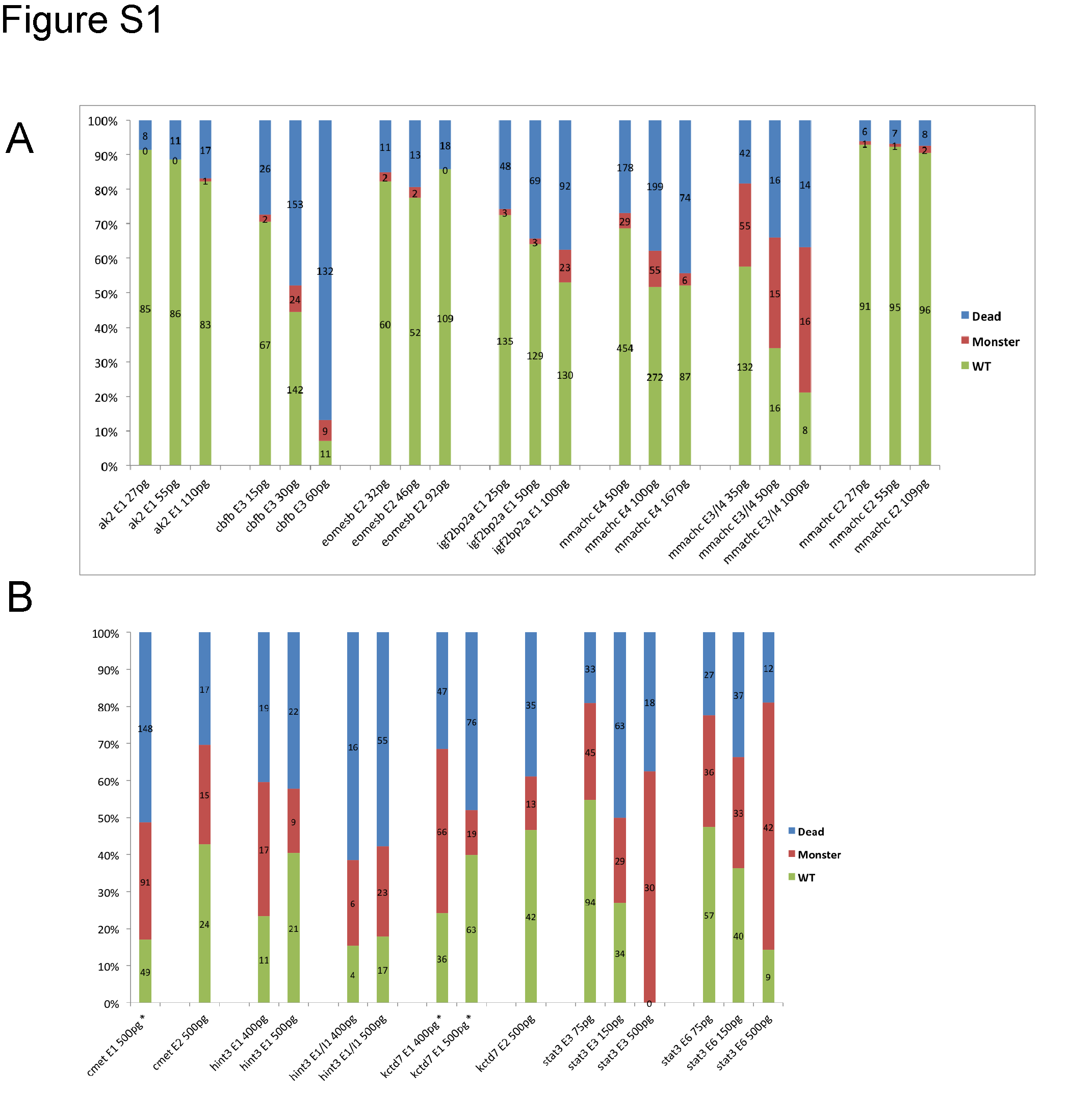

Supplement: Figure S1 — Toxicity data for CompoZr ZFNs (A) and CoDA ZFNs (B). ZFN ID's and injected doses are listed on the X-axis and percentage of normal (WT = green), deformed (Monster = red) and dead (blue) embryos at 24 hpf are shown on the Y-axis. Numbers in the bar graphs denote the actual numbers of embryos in each category. (TIF) [file pone.0057239.s001.tif]
